# Immediate Autotransplantation of Mature Third Molars to Bilateral Mandibular First Molars With Large Periapical Lesions: A Case Report

**DOI:** 10.1002/ccr3.70581

**Published:** 2025-07-13

**Authors:** Ailimaierdan Ainiwaer, Ling Wang

**Affiliations:** ^1^ Outpatient Department of Oral Surgery Research Institute of Stomatology of Xinjiang Uygur Autonomous Region, the First Affiliated Hospital of Xinjiang Medical University Urumqi China

**Keywords:** autogenous tooth transplantation (ATT), concentrated growth factor (CGF), mature third molars, periapical lesions

## Abstract

Previously reported protocols suggested that the transplantation of teeth should be delayed if the recipient site presents large periapical lesions. The presented case is a pioneering attempt to conduct mature third molar transplantation immediately after the extraction of mandibular first molars accompanied by large periradicular lesions with the guidance of computer‐designed 3D‐printed replicas and with the utilization of concentrated growth factor (CGF) so as to avoid the use of synthetic bone substitutes and secondary surgical procedures. A 24‐year‐old female patient in generally good health was presented with pain and swelling of bilateral mandibular first molars accompanied by a sinus tract. Cone‐beam computed tomography (CBCT) revealed 16 * 10 mm and 13 * 7 mm low density, respectively, in the periapical area of #46 and #36. After confirmation of the feasibility of transplanting the mandibular third molars to the first molars with the evaluation by CBCT, patient's consent was obtained. Based on patient's CBCT, individual 3D‐printed replicas of the mandibular third molars were fabricated and CGF was collected by centrifugation. Autogenous tooth transplantation (ATT) was performed immediately after the removal of the first molars and curettage of periapical lesions. The periapical bone defect was filled with CGF membranes. After the surgery, healing conditions were monitored. At 18‐month follow‐up, both transplanted teeth presented with stable healing without any clinical complications. Radiographically, periapical new bone healing at both recipient sites was observed. This case indicates that immediate transplantation of mature teeth to recipient sites with large periapical lesions can be a viable treatment option and can lead to optimistic results.

**Summary**

- Immediate autotransplantation of mature third molars to recipient sites with large periapical lesion can be a viable treatment option and can lead to optimistic results.
- Some standards of autogenous tooth transplantation may be reconsidered regarding the treatment options on similar cases.

## 1 Introduction

Autogenous tooth transplantation (ATT) is the repositioning of an erupted, partially erupted, or non‐erupted autologous tooth from its original location to another site within the same individual. The history of modern tooth autotransplantation can be traced back to 1950s [1]. With a better understanding of alveolar–periodontal microenvironments, the development of atraumatic surgical techniques during the surgery, along with careful postoperative care, the success rate and long‐term survival of ATT went through affirmative changes over the long course of clinical application, indicating ATT is a viable option for tooth replacement for carefully selected patients [2, 3].

There are several factors which influence the success rate of autotransplantation such as age, plaque control, the stage of root development, the morphology of the donor tooth, extra‐oral time, vitality of the cells in the periodontal ligament (PDL), the shape, vascularity and integrity of the recipient socket, fixation methods, and postoperative maintenance along with systemic antibiotics and endodontic treatments [2, 3, 4, 5]. Among which, preservation of intact and active PDL and Hertwig's root sheath on the root surface is the key factor for successful autotransplantation [3]. In order to achieve this, reduction of trauma following minimally invasive procedures and shortening the extra‐oral time of the donor tooth is very necessary. With the guidance of 3D‐printed replicas, the extra‐oral time of donor tooth can be reduced significantly improving the success rate of ATT [6, 7].

Most researches stated that the recipient site must be free from any pathological activity in order to achieve successful transplantation [4, 8, 9]. Some scholars recommend that the conduction of ATT should be postponed 8–12 weeks or even a longer period of time after the extraction of the tooth in the recipient site and total curettage of lesions when the recipient site is accompanied by large inflammation [4, 10]. However, this procedure increases the operation time and postpones the repair of dentition, and more importantly, the patient has to undergo a second surgery with pain and cost. A pioneering report indicated the possibility of immediate immature third molar transplantation into lesion‐associated recipient sites after being managed appropriately [11]. Yet, to the best of our knowledge, there are no relevant studies considering immediate mature third molar transplantation in cases with large periapical lesions in the recipient site.

The presented case is a pioneering attempt to conduct immediate mature third molar transplantation to the recipient site accompanied by a large periapical lesion with the guidance of 3D‐printed replicas and the utilization of concentrated growth factor (CGF), so as to avoid the use of synthetic bone substitutes and secondary surgical procedures. In this case report, we describe a 24‐year‐old patient who underwent immediate ATT after the removal of bilateral mandibular first molars and the curettage of periapical lesions. The treatment results of the case followed up for 18 months are reported as follows.

## 2 Case History/Examination

**FIGURE 1:**
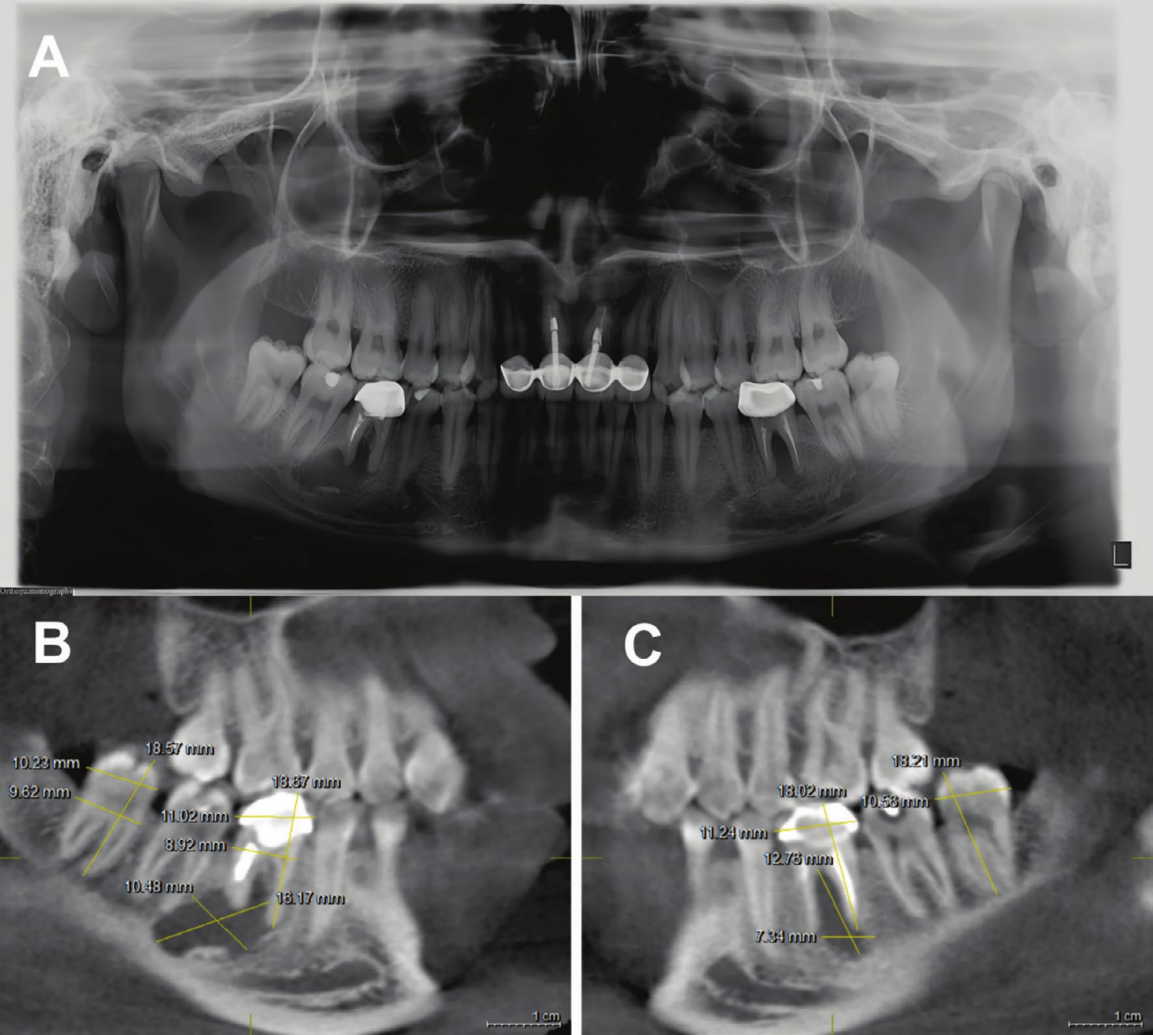
Preoperative radiographic conditions of recipient sites (#36, 46) and donor teeth (#38, 48). (A) Curved panoramic view. (B, C) Sagittal plane view.

A female patient, aged 24, was presented to the Department of oral surgery clinic, the First Affiliated Hospital of Xinjiang Medical University, with pain and swelling in the bilateral mandibular posterior teeth area of the lower jaw. The affected teeth was treated with root canal tratment (RCT) and repaired with a porcelain crown in the outer hospital 5 years ago. Clinical examination showed that there was a fistula in the left posterior buccal gingiva, with slight swelling and palpation pain. Preoperative examination results of routine blood test showed no obvious abnormalities. The cone‐beam computed tomography (CBCT) were shown in (Figure 1A–C), showing (16 * 10) mm and (13 * 7) mm low density, respectively, in periapical area of #46 and #36. The difference between the width and length of the recipient teeth (#36 and #46) and the donor teeth (#38 and #48) were within 2 mm.

## 3 Methods (Investigations and Treatment)

Due to the scope of low density area on the right side was larger than the left side, the surgical procedure of the patient was to transplant the right impacted tooth (#48) to the right mandibular first molar (#46) first, and then the left side after 2 months. Before operation, the patient was informed about the potential benefits and risks of surgery, as well as alternative treatment options, and volunteered to participate in and signed an informed consent. The protocol of this study was consistent with the ethical guidelines of the Declaration of Helsinki.

**FIGURE 2:**
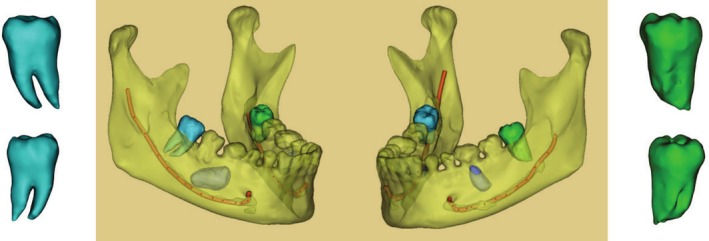
Preoperative computer designed 3D images of the mandible and the donor teeth (#38, 48).

The data obtained by the CBCT was converted into DICOM format, and then imported into MIMICS software and 3D printer to replicate and print the resin tooth model (Figure 2). Acrylic models were sterilized by ethylene oxide before surgery, aseptically packed and ready for use.

**FIGURE 3:**
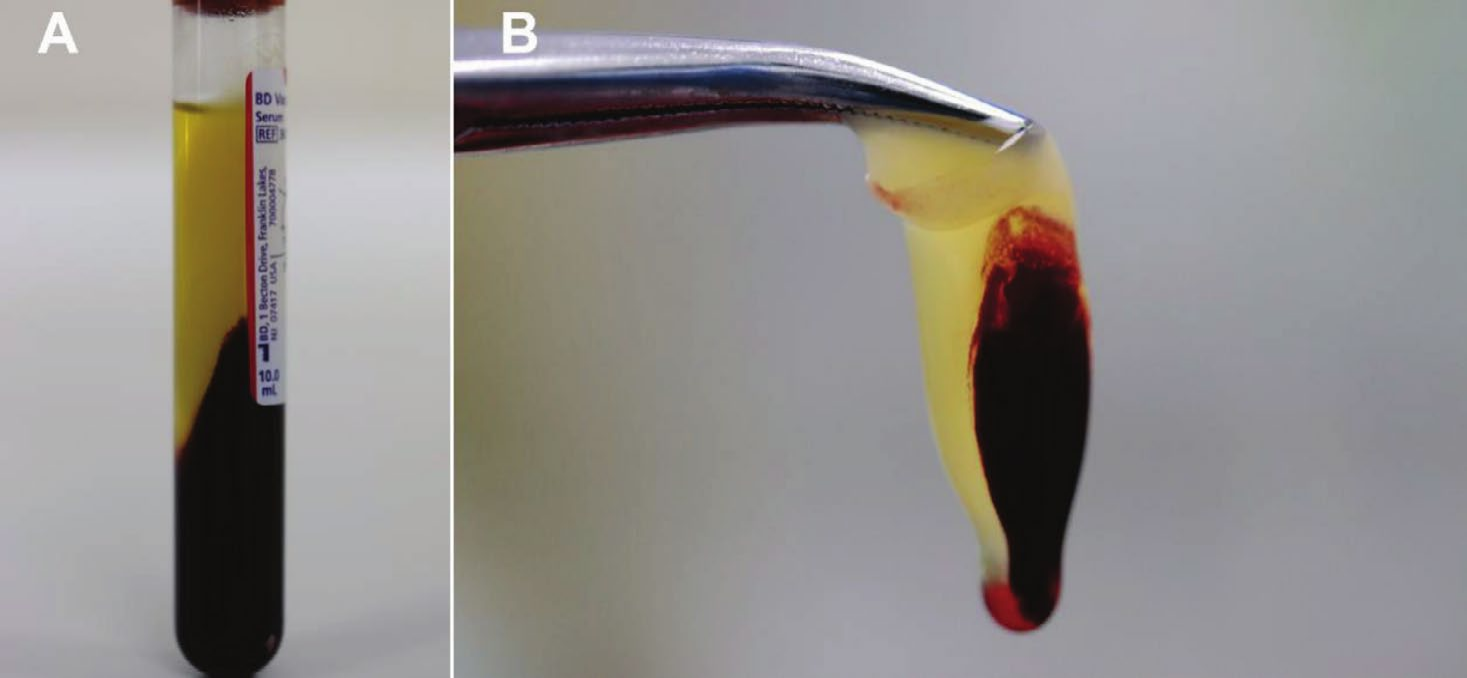
The concentrated growth factor (CGF) obtained by centrifugation. (A) Blood tubes after centrifugation and the three layers. (B) CGF separated with sterile scissors.

Venous blood sample was collected from the patient into a sterile 10‐mL blood tube (vacuum negative pressure inside, tube wall coated with silica particles, and without anticoagulant) before the operation. The tube was placed in a Medifuge centrifuge (Silfradent, Italy) and followed by centrifugation at the program of 30‐s acceleration, 2 min 2700 rpm, 4 min 2400 rpm, 4 min 2700 rpm, 3 min 3000 rpm, and 36 s deceleration. After centrifugation, the CGF layer, which was the second of the three layers (Figure 3A), was separated with a sterile scissor for intraoperative use (Figure 3B).

**FIGURE 4:**
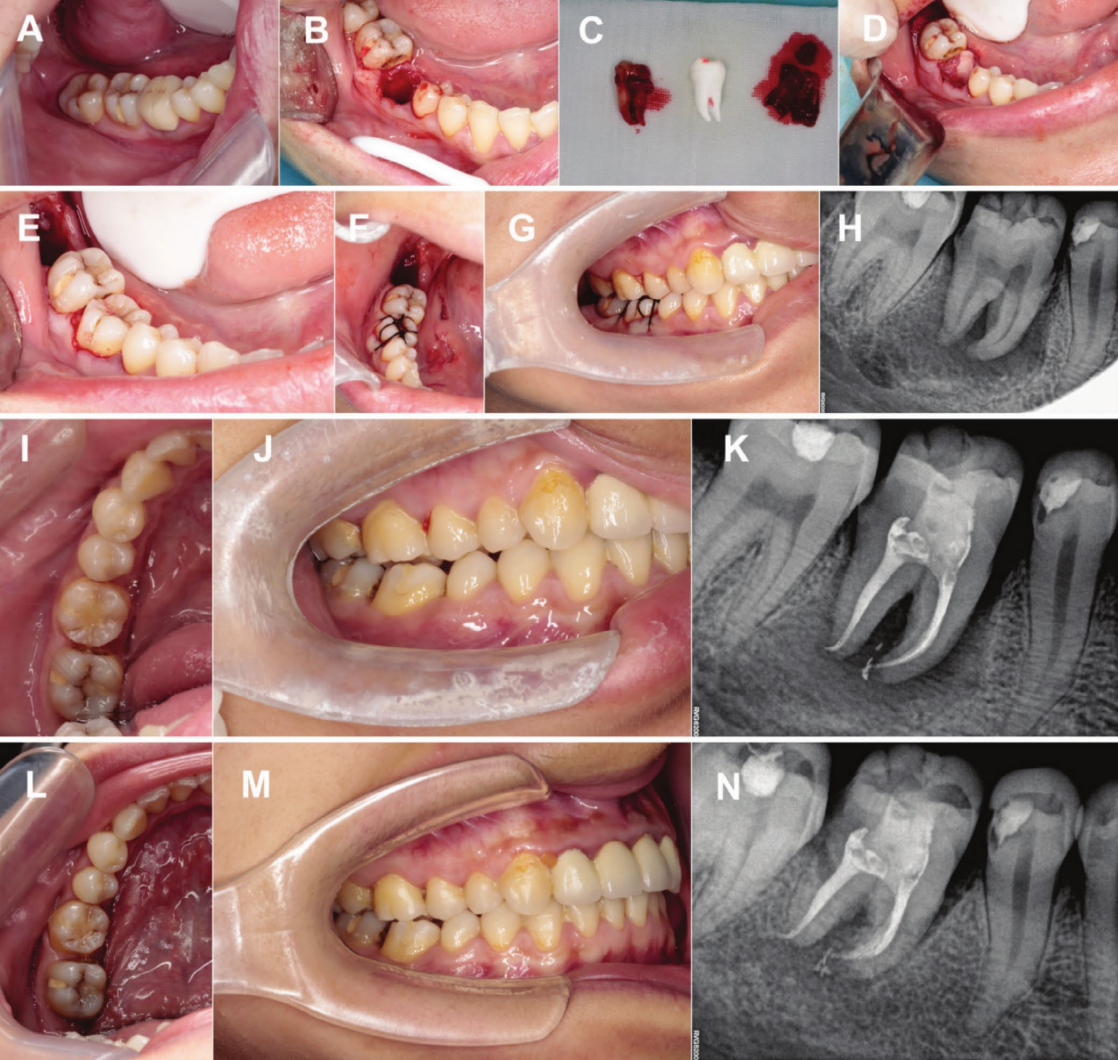
Surgical procedure of #48 transplanted into fresh socket of #46 and postoperative follow‐up. (A) Compromised first molar and impacted tooth with vertical eruption. (B) Prepared fresh socket of the first molar after extraction. (C) The donor tooth after extraction and its 3D replica. (D) CGF placed in the extraction socket. (E) Placement of the donor tooth (#48) to the recipient site (#46). (F) The autotransplanted tooth was sutured to the gingiva in 8‐like shape. (G) The occlusal relationship of the autotransplanted tooth (#46) immediately after operation. (H) Periapical X‐ray examination immediately after operation. (I–K) Adjacent and occlusal relationship and periapical X‐ray of the autotransplanted tooth (#46) at 3 months follow‐up. (L‐N) Adjacent and occlusal relationship and periapical X‐ray of the autotransplanted tooth (#46) at 18 months follow‐up.

**FIGURE 5:**
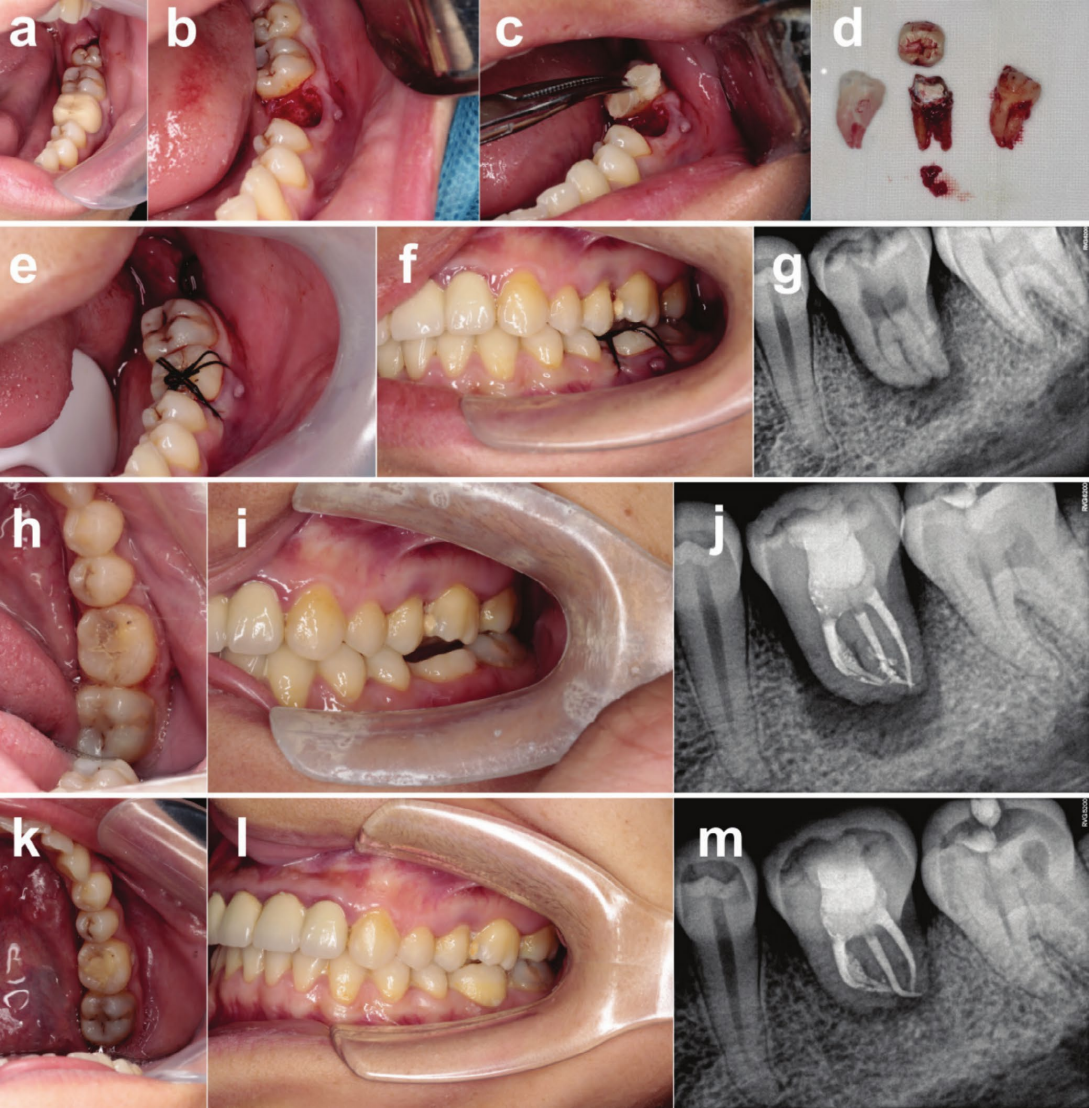
Surgical procedure of #38 transplanted into fresh socket of #36 and postoperative follow‐up. (a) Compromised first molar and impacted tooth with vertical eruption. (b) Prepared fresh socket of the first molar after extraction. (c) CGF placed into the extraction socket. (d) The donor tooth after extraction and its 3D replica. (e) Placement of the donor tooth (#38) to the recipient site (#36) and suturation. (f) The occlusal relationship of the autotransplanted tooth (#36) immediately after operation. (g) Periapical X‐ray examination immediately after operation. (h–j) Adjacent and occlusal relationship and periapical X‐ray of the autotransplanted tooth (#36) at 1‐month follow‐up. (k–m) Adjacent and occlusal relationship and periapical X‐ray of the autotransplanted tooth (#36) at 16 months follow‐up.

The patient was treated with antibiotics 1 day before and 3 days after surgery. The oral cavity was cleaned with compound chlorhexidine with the concentration of 0.12%, and the injection site of anesthesia and operation area were disinfected with iodophor cotton balls. After local anesthesia was performed with 2% lignocaine in 1: 200000 dilution adrenaline, the compromised molar was extracted atraumatically. Preliminary preparation of the alveolar socket was conducted by removing the intra‐alveolar septa with burs to convert the extraction socket from a double‐root shape into a single‐root shape. The alveolar bone in the periapical area of the recipient site was then carefully enucleated by using bone curettes. The 3D model of the donor tooth was used to guide the modification of the alveolar socket until it was completely in place. The adjustment of occlusal position between the recipient teeth (#36 and #46) and the opposite teeth (#26 and #16) were adjusted before the extraction of donor teeth (#38 and #48) with the guidance of 3D replicas. Then the CGF membrane with certain elasticity and adhesion was placed in the alveolar fossa of the recipient site to make the CGF membrane completely cover the bone defect. After minimal invasive extraction, the impacted tooth was immediately transplanted into the recipient socket to check whether they reached the ideal position (the extra oral time of both donor tooth were less than 60 s). When further adjustment was needed, the donor tooth was placed in the sterile saline. The donor tooth was put in place by using finger pressing without touching the root. After transplantation, the tooth were fixed with 8‐shaped cross suture and periapical radiograph was taken immediately after the surgery as a baseline reference (Figures 4A–H and 5a–g).

## 4 Conclusion and Results (Outcome and Follow‐Up)

After surgery, the patient was instructed about appropriate hygienic‐dietary regimen and treated with antibiotics for 3 days. Sutures were removed 7 days later. RCT was conducted 3 weeks postoperation. The patient was scheduled for visit at 3, 6, 12 months, and once every 6 months after 1 year. Clinical parameters including periodontal probing, mobility, percussion, and occlusal status were examined every visit and periapical X‐ray was conducted (Figures 4K,N and 5j,m). The autotransplanted teeth were in good periodontal health and functional status during the 18 months follow‐up (Figures 4I–N and 5h–m). We noticed that both transplanted teeth established occlusal contact with opposing teeth at the last follow up and the fistula was replaced with normal gingival tissues. Periapical X‐ray showed healing of the alveolar bone at the apical zone and the transplanted teeth showed normal periodontal space along with normal lamina dura with no evidence of ankylosis, internal and external root resorptions or periapical pathology. During the follow‐up, the patient had no complaints and showed satisfaction toward the treatment outcome.

## 5 Discussion

This case report presents successful immediate autotransplantation of mature third molars into mandibular first molar sites with large periapical lesions. Although few similar cases were found in the literature, these cases described ATT of immature donors to sites with inflammation. Erdem and Gümüşer [11] reported successful outcome of simultaneous autotransplantation of 12 immature third molars to mandibular first or second molar sites with chronic periapical lesions in patients aged 15–21. Besides, a case report described successful delayed autotransplantation in a case with inflammation in recipient site: Arbel et al. [10] carried out a delayed autotransplantation of immature third molar to a mandibular molar with over 15 mm diameter of periradicular lesion in periapical area 9 weeks after removing the molar and curetting the lesions on a 19‐year‐old patient and observed good healing of both transplanted tooth and alveolar bone after 1 year follow‐up. However, immature teeth, especially when the root developed to 1/2–3/4 of length (or stage3‐stage4 according to classification of Moorrees et al. [12]) can achieve highest ATT success rate up to 100% because immature teeth possess wider PDL layer than mature teeth, which enables the immature teeth to possess more regenerative capacity along with significant potential for revascularization and reconstruction of pulpal tissues [4, 13, 14, 15]. Due to the lack of research with larger sample sized cases conducted with mature third molars, the specific procedures and outcomes of such conditions are obscure. The present case highlights the possibility of immediate ATT of third molars with complete root formation into recipient sites with large periapical lesions.

It is well known that during the healing process of an autotransplanted tooth, the PDL on the root surface plays a crucial role in periodontal ligament healing and alveolar bone remodeling [2, 3, 4]. Preservation of intact PDL on the root surface can be achieved by atraumatic donor‐site extraction during the surgical process and reducing the extra‐oral time of the donor tooth. Andreasen [16] reported that 80% of the cases presented normal periodontal ligament healing when extra‐oral times were limited to within 18 min. The application of 3D‐printed replicas increases the ease of surgery, and the extra‐oral time can be reduced to within 1 min, significantly increasing the success rate of ATT [2, 6, 7, 15]. With the guidance of 3D‐printed replicas, the extra‐oral time of both donor teeth in our case was less than 1 min; we believe that the success of this case was closely related to the extremely short extra‐oral time of the donor teeth.

As the newest generation of autologous platelet concentrate, CGF has been reported to induce the osteogenic differentiation of mesenchymal stem cells, promote the proliferation and migration of osteoblasts and osteocytes, inhibit bone resorption, and accelerate the regeneration of bone tissues [5, 17, 18]. Many clinical investigations found that CGF can promote the healing of hard and soft tissues and reduce postoperative pain, and it has been used extensively in the field of dentistry [5, 6, 18]. In our previous study, we discovered that the application of CGF to recipient sites with chronic periapical lesions (diameter less than 1 cm) can accelerate the regeneration of the alveolar bone and healing of inflammation after tooth transplantation, greatly shortening the healing period and reducing postoperative pain in the early stages of healing [6]. In this case, we monitored new bone formation at the site of the alveolar lesion at 6‐month visit, and both transplanted teeth established occlusal contact with opposing teeth at the last visit compared with the occlusal space immediately after the operation. This may be because the rapid bone healing is attributed to the occlusal elevation of the transplanted teeth.

The main challenge of this case is the existence of large chronic periapical inflammations in both recipient site. If the range of inflammation in the periapical area is large, the overall healing time takes up to 2 years and the risk of healing failure and recurrence is relatively high while smaller defects takes about 1 year [19, 20]. After ATT was conducted, the percent bone change significantly increases during the first 3 months and during this period, the operation site should be free of inflammation [21]. Unlike immature teeth, success rate of autotransplanted mature third molars treated with RCT were higher than those not treated with RCT [2, 15, 22]. In order to ensure smooth progress of this case, on the one hand, we removed all the chronic inflammatory tissues in the recipient site thoroughly during operation by delicate curettage and irrigation, and on the other hand, we conducted RCT 3 weeks postoperation to avoid pulp infection.

Although long‐term follow‐up is preferred, the current status of both transplanted tooth was in favorable prognosis with successful healing of both hard and soft tissues, with good chewing function and patient satisfaction without complaints. However, due to the limited number of cases and short observation time, in‐depth study is required for large‐sample, multicenter, randomized controlled clinical study in the later stage.

## 6 Conclusion

Immediate autotransplantation of mature third molars to replace mandibular first molars with large periradicular lesions with the guidance of computer‐designed 3D‐printed replicas and with the utilization of CGF can be a valid treatment option. Further studies with a longer period of follow‐up and larger sample size are warranted.

## Author Contributions

**Ailimaierdan Ainiwaer:** conceptualization, operator of the surgery,data curation, formal analysis, methodology, visualization, writing – original draft, writing – review and editing. **Ling Wang:** conceptualization, methodology, visualization, writing – review and editing.

## Ethics Statement

Ethics approval is not required for de‐identified single case reports based on institutional policies.

## Consent

Written informed consent was obtained from the patient to publish this report in accordance with the journal's patient consent policy.

## Conflicts of Interest

The authors declare no conflicts of interest.

## Data Availability

Data sharing is not applicable to this article as no datasets were generated or analyzed during the current study.
